# Case report: Local cryoablation combined with pembrolizumab to eliminate lung metastases from ovarian clear cell carcinoma

**DOI:** 10.3389/fimmu.2022.1006500

**Published:** 2022-11-10

**Authors:** Liangliang Meng, Zhongliang Zhang, Xiao Zhang, Xiaobo Zhang, Yingtian Wei, Bin Wu, Xiaodong Xue, Xin Zhang, Xiaofeng He, Yueyong Xiao

**Affiliations:** ^1^ Department of Radiology, the First Medical Center, Chinese PLA General Hospital, Beijing, China; ^2^ Department of Radiology, Chinese PAP Hospital of Beijing, Beijing, China

**Keywords:** ovarian cancer, lung metastases, cryoablation, PD-1, pembrolizumab

## Abstract

Ovarian clear cell carcinoma has a high recurrence rate with poor prognosis and is generally not sensitive to conventional platinum-based chemotherapy. Its less frequent occurrence of mutations such as BRCA limited the targeted therapies. Immunotherapy is not currently recommended as a first-line agent for ovarian cancer, and most patients are not yet able to benefit from it. Cryoablation can be used to treat solid systemic tumors, including ovarian cancer metastases, and can produce a limited anti-tumor immune response. The anti-tumor effects of cryoablation combined with immunotherapy have not been adequately confirmed. This study reports a case of a patient with ovarian clear cell carcinoma who underwent conventional adjuvant chemotherapy after initially surgical resection of the tumor. Unfortunately, cancer recurred and metastasized to the abdominal wall. After a series of painful chemotherapy and a second surgery, the cancer was still not effectively controlled, and the patient developed extensive metastases in the lung. The patient’s PD-L1 expression level also did not support solo immunotherapy. We pioneered the use of cryoablation to first eradicate the most significant lesion in the upper lobe of the left lung and then combined it with the PD-L1 inhibitor pembrolizumab to treat the patient with immunotherapy, which resulted in the complete eradication of the other multiple metastases in the lung and saved the patient’s life. Although the precise mechanism of action has not yet been explored, we have reason to believe that the combination of cryoablation and immune checkpoint inhibitor has a powerful synergistic anti-tumor effect, which is yet to be confirmed by more basic research and clinical applications in the next step.

## Introduction

Epithelial ovarian cancer (EOC), referred to as ovarian cancer, is a heterogeneous group of diseases with different genomic characteristics, prone to chemotherapy resistance resulting in a high recurrence rate and poor prognosis, with the highest death rate among gynecological malignancies (1, 2). Ovarian clear cell carcinoma (OCCC) is the second most common histological type of EOC (1, 3). Early-stage OSCC should be treated with complete tumor staging surgery, including total hysterectomy, adnexal resection, and abdominal lymph node dissection. Patients with advanced OCCC have a worse prognosis than other histological subtypes of ovarian cancer. Clinical diagnosis and treatment are challenging, and targeted clinical guidelines for diagnosis and treatment are lacking. Chemotherapy is the conventional adjuvant treatment for OCCC after surgery. Still, its effect is not apparent, especially for the resistance to platinum-based chemotherapy drugs (4)。Immunotherapy, especially immune checkpoint inhibitors, is currently a hot topic in oncology research. Clinical trials have been conducted for recurrent or metastatic OCCC to validate the anti-tumor activity and safety of immunotherapy with PD-1 antibody (pembrolizumab) in 376 patients with recurrent ovarian cancer. The effectiveness rate was only 8% in all patients and 15.8% in 19 patients with OCCC. Most of the patients did not benefit from it (5).

Cryoablation is to place a probe in the center of the tumor to induce tumor tissue necrosis and apoptosis through intracellular ice crystal formation and cell destruction caused by ultra-low temperature (6). For all primary solid tumors and metastatic tumors at all sites, imaging-guided cryoablation can destroy tumors with minor trauma and faster recovery and can be applied to multiple lesions simultaneously or repeatedly (7, 8). Cryoablation has also been shown to promote a limited anti-tumor immune response and may also have a powerful synergistic anti-tumor effect with immunotherapy (9–11).

This study reports a patient with recurrent, metastatic ovarian clear cell carcinoma whose tumor was not effectively controlled after a series of chemotherapy and second surgery. After our pioneering use of cryoablation to eradicate a more significant lesion in the left lung, the patient was subsequently treated with immunotherapy in combination with the PD-L1 inhibitor palivizumab, which destroyed the other multiple lesions in the lung and saved the patient’s life.

## Patient description

This retrospective study was approved by the Ethics Committee of the Chinese PLA general hospital. Written informed consent was provided for this study by the patient. The patient was a 48-year-old female who underwent surgical resection after evaluation for a left ovarian tumor discovered in July 2017. Intraoperative evaluation after opening the abdomen resulted in surgical resection of the entire uterus and adnexa, the appendix and part of the greater omentum, and pelvic lymph nodes were cleared. The postoperative pathological diagnosis was clear cell carcinoma of the left ovary, and cancer cells were seen in a section of the left fallopian tube. In contrast, no cancer cells were seen in the greater omentum and appendix.

After the surgical procedure, the patient was given 6 cycles of systemic adjuvant chemotherapy based on the consultation with the surgeon and medical oncologist. The chemotherapy protocol was Lipoxin plus carboplatin. After chemotherapy, the patient was followed up regularly every six months, and no recurrence or metastasis was detected.

**Figure 1:**
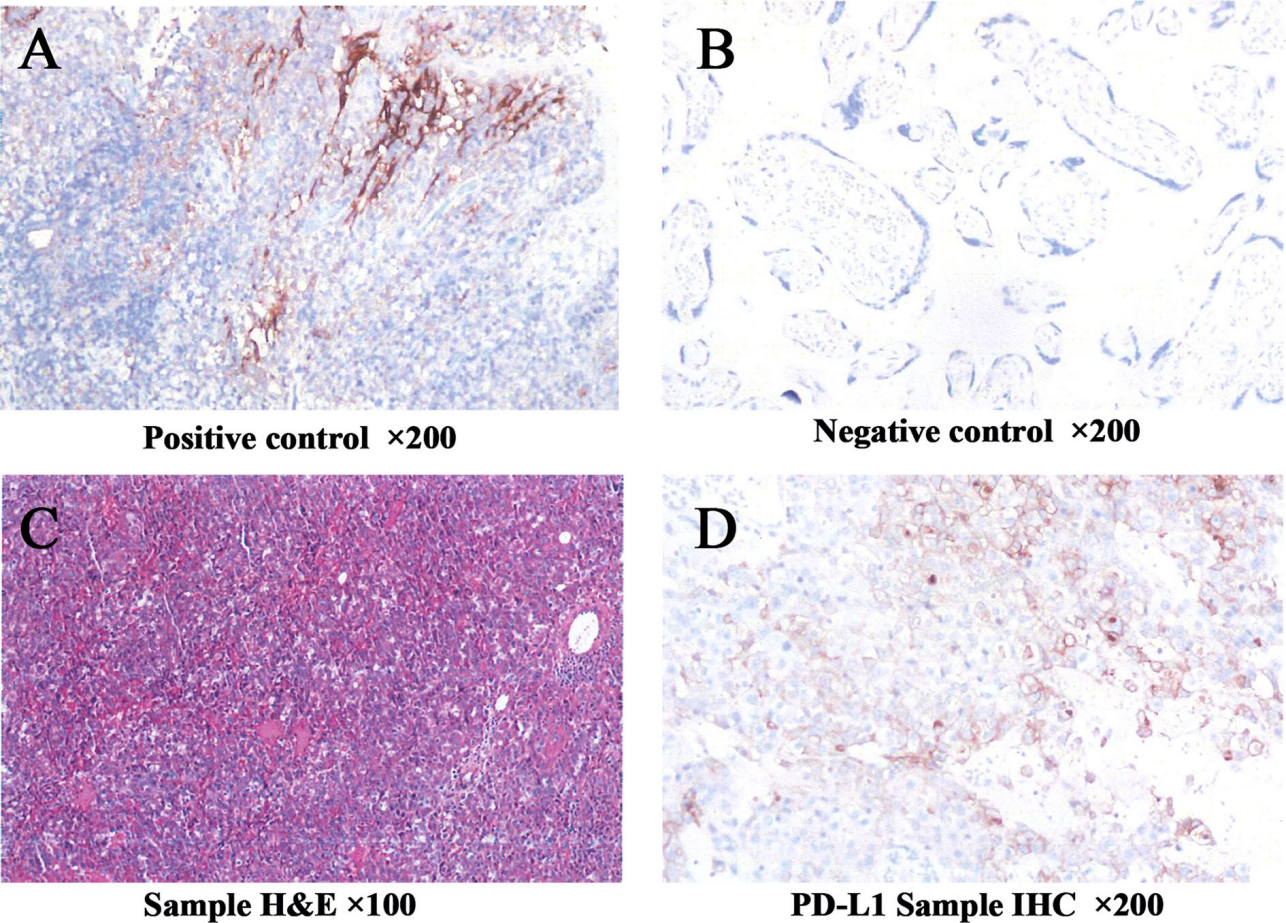
Immunohistochemistry showed that PD-L1 expression in the tumor tissue was 5% for TPS and 10 for CPS **(A–D)**. TPS, tumor proportion score; CPS, combined positive score.

Three years later, in November 2020, the patient was found to have a left abdominal wall mass on follow-up examination, and the size of the lesion was found to be about 3.3*1.9 cm on a CT scan. Multiple nodules were also present in the bilateral lungs, suggesting significant tumor progression and possible systemic metastasis. After consultation and with the family’s consent, surgical resection was performed for the left abdominal wall mass, and the pelvic metastatic lymph nodes were also cleared. The postoperative pathological diagnosis of the left abdominal wall mass and the metastatic pelvic lymph nodes was: ovarian clear cell carcinoma metastasis. To further clarify the nature of the tumor and its responsiveness to immunotherapy and targeted therapy, and immunohistochemical analysis of the metastatic tumor tissue was performed. The results showed that BRCA1(-), PD-L1 TPS=5%, CPS=10, TMB 3.91Muts/Mb medium (lower than 67% of ovarian cancer patients), and microsatellite stable (**Figure 1**). EBV test result was negative. The above results suggest that immune checkpoint inhibitors of the PD-1/PD-L1 pathway may have some effect on this patient. Still, the effect is limited and cannot be used as a basis for single-agent application according to clinical guidelines. After the physician informed the family of the circumstances, the family decided to withhold immunotherapy after communication with the patient. To stop the further spread of the tumor, the medical oncologic department recommended continuing chemotherapy. Two cycles of chemotherapy were administered from November 2020 to February 2021, and the treatment protocol was Tysol plus Boldin. During the chemotherapy period, there was an III° decrease in neutrophils, CT scan evaluation of multiple foci in both lungs was larger than before, and the efficacy was assessed as PD. After a multidisciplinary team discussion, the chemotherapy regimen was changed to CPT11 plus gemcitabine for six cycles, with the last chemotherapy on June 10, 2021. CT examination after chemotherapy showed that multiple solid nodules of varying sizes were still seen in both lungs, with the larger one measuring about 20×12 mm. During the long period of chemotherapy, the patient suffered a lot of pain and mental stress.

**Figure 2:**
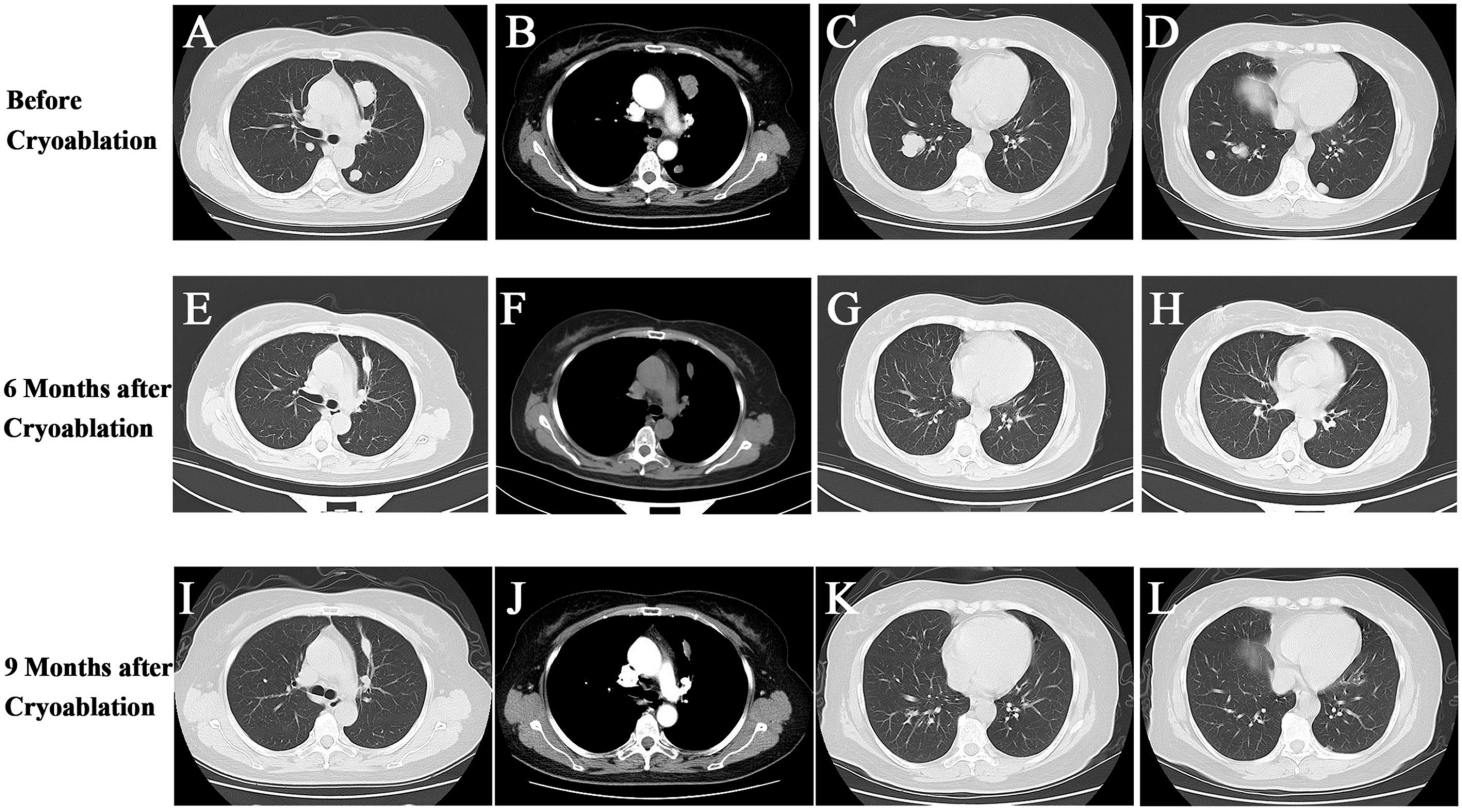
1 CT follow-up images of the patient before and after cryoablation. **(A–D)** Before cryoablation of the patient, multiple masses and nodules of variable size were seen in the left upper lobe and lower lobe of both lungs. **(E–H)** Six months after cryoablation of the more significant lesion in the left upper lobe, the frozen area was seen to be smaller and firmer; the remaining multiple lung lesions had largely disappeared. **(I–L)** Nine months after cryoablation, only scarring was seen in the cryoablation area, and no lesions were seen in both lungs.

After chemotherapy, the patient took oral Chinese medicinal agents continuously for about 6 weeks to modulate her health. The exact names and ingredients of the drugs are unknown. Later, the patient sought further consultation and treatment for multiple lung foci and came to our institution. After admission, CT showed that multiple masses and nodules of different sizes were visible in both lungs of the patient. The larger one was about 33 mm in diameter, and all were considered to be metastases in combination with medical history (**Figures 2A–D**).

**Figure 3:**
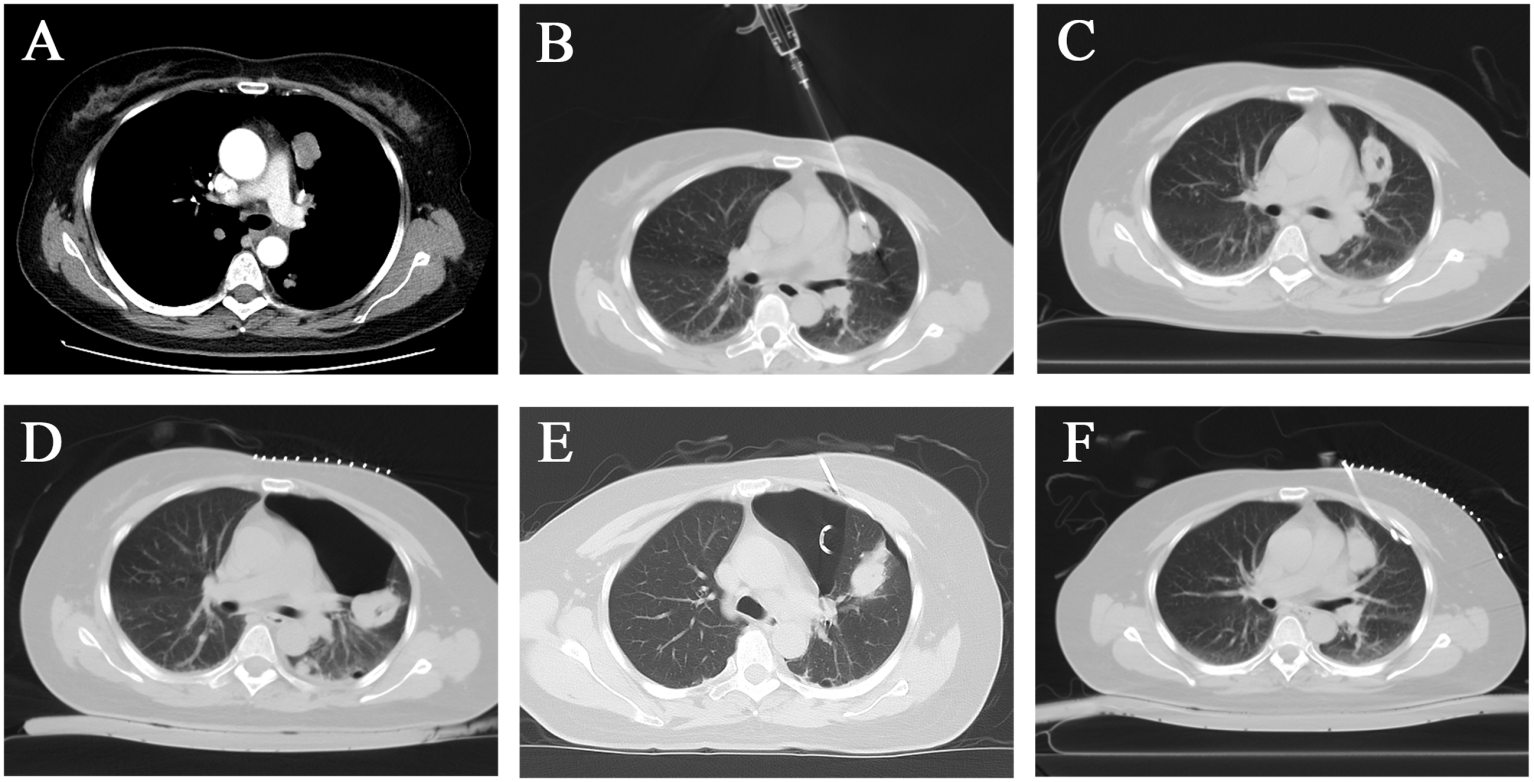
After CT-guided percutaneous biopsy, the patient developed pneumothorax and decreased oxygen saturation, which was treated with CT-guided tube drainage. **(A)** A left upper lobe mass with significant enhancement. **(B)** The needle slot of the 18G semi-automatic puncture needle was located in the center of the tumor. **(C)** No significant postoperative bleeding or pneumothorax was observed. **(D)** Pneumothorax was caused by the harsh coughing of the patient. **(E)** The patient was treated with a chest tube for drainage. **(F)** The pneumothorax had utterly disappeared after 1 week.

To determine the pathological type of the multiple pulmonary tumors to guide the following treatment, we first selected the larger lesion in the left upper lobe of the lung for CT-guided percutaneous puncture biopsy after the routine preoperative examination upon admission (**Figure 3A**). Three strips of tumor tissue were obtained and sent for testing after puncture with a BD 18G co-axial biopsy needle (Bard Peripheral Vascular Inc, USA) (**Figure 3B**). After a minimally invasive biopsy, the patient did not experience significant bleeding or pneumothorax on the immediate postoperative CT scan (**Figure 3C**). Approximately 6 hours after surgery, the patient developed a sharp cough, followed by breath-holding and decreased oxygen saturation. CT scan showed a left-sided pneumothorax was developed, closed chest drainage was given, and the tube would be removed after the gas was drained (**Figures 3D–F**).

The pathology results showed adenoidally arranged heterogeneous cells in the punctured tissue, which, together with the history and immunohistochemical findings, was consistent with ovarian clear cell carcinoma metastasis. The results of immunohistochemistry were TTF-1(-), p16 (individual +), p53 (+90%), PAX-8 (+), WT-1 (local weak +), ER (-), PR (-), NapsinA (partial +), CK7 (+), Ki-67 (+30%).

Because there were too many lesions in the lungs to perform surgical resection, and for more extensive lesions, radiofrequency or microwave may not easily inactivate the lesions completely. Finally, after evaluation of the patient, CT-guided percutaneous left lung lesion argon-helium cryoablation was planned to destroy the larger tumors while clarifying the pathology to guide the following treatment plan. Although the gas in the thoracic cavity was completely drained, we decided to keep the chest drain until after the ablation to prevent pneumothorax recurrence during and after the cryoablation procedure.

**Figure 4:**
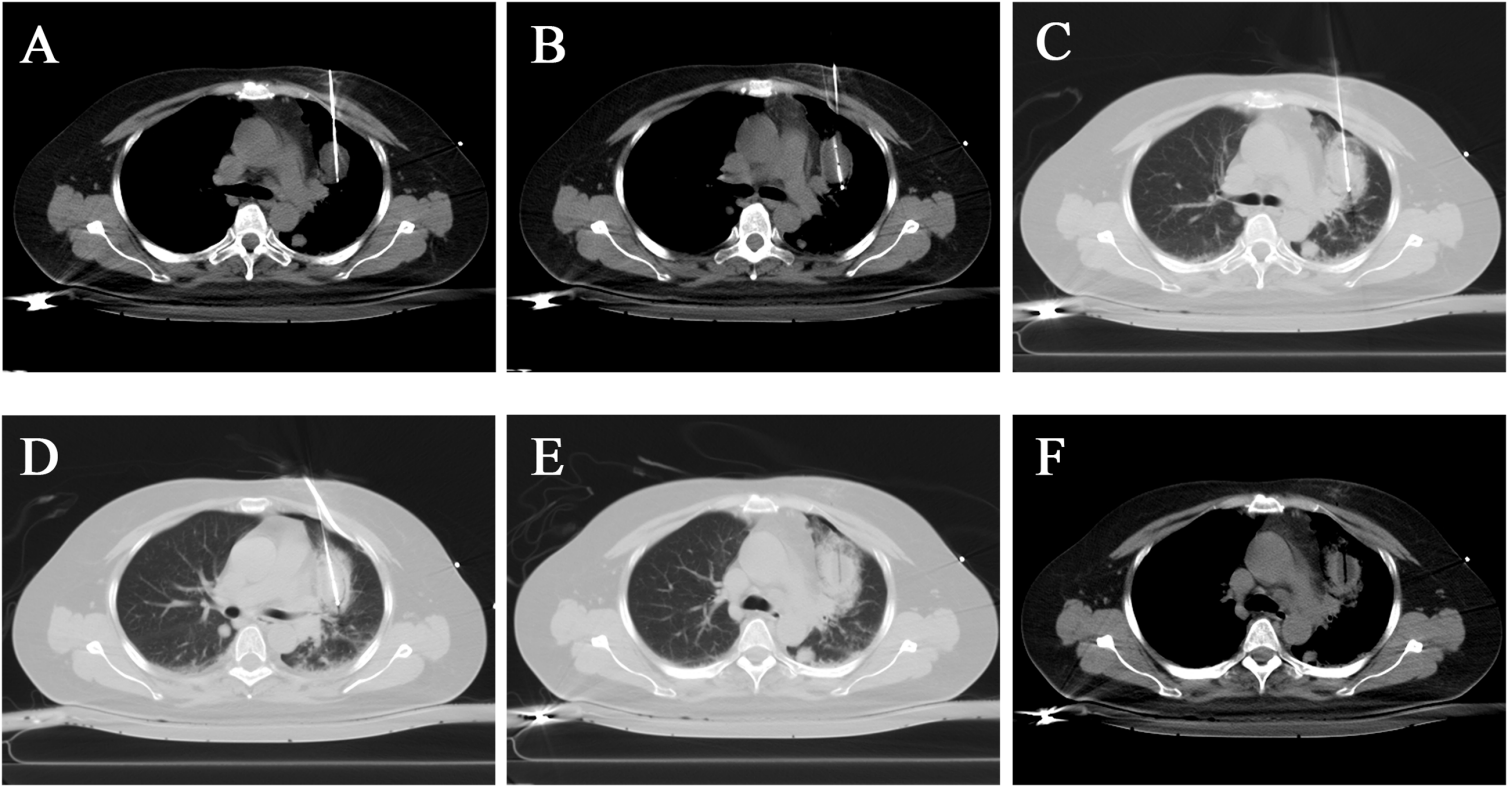
Cryoablation of a larger lesion in the upper lobe of the left lung. **(A, B)** Two probes were placed in different tumor parts, and cryoablation was performed on the tumor. The mediastinal window visualized the tumor filled with a low-density ice ball. **(C, D)** The lung window showed a ring-like increase in lung tissue density around the tumor. **(E, F)** After the cryoablation was completed, the cryo-probes were removed, and residual needle tracts were visible.

Cryoablation treatment procedure: The patient was supine on the CT examination table, with routine monitoring and establishing intravenous access. After the CT scan was performed, the skin was marked with the puncture site with a routinely regional disinfected and sterile towel laid, 1% lidocaine was used for local anesthesia, and two 17G cryoprobes were percutaneously punctured into the left upper lobe of the lung under the CT-guidance, two freeze-ablation cycles were performed (**Figures 4A–D**). CT scans were performed at 5-minute intervals during the treatment to determine the ice ball’s location and extent. The CT scan was performed again after removing the probe to confirm the treatment’s effectiveness and exclude complications, including bleeding. No abnormality was found in the postoperative CT scan (**Figures 4E, F**). Postoperatively, the patient was given a flurbiprofen ester injection and pethidine hydrochloride injection as a symptomatic treatment for pain. Because of the large lesion, the patient was given hydration and alkalinization of urine treatment, and liver and kidney function were closely monitored to prevent liver and kidney failure. Preoperative treatment was given to avoid infection. Postoperatively, symptomatic treatment such as analgesia and anti-inflammation was given.

Considering the immune effect of cryoablation and the synergistic anti-tumor effect between cryoablation and immunotherapy, combined with the patient’s previous immunohistochemical results, we believed that cryotherapy combined with immunotherapy might have a significant therapeutic effect on tumors in other sites of the body. Therefore, after communication with the patient and family, systemic immunotherapy was subsequently administered. The intravenous infusion drug was PD-1/PD-L1 pathway inhibitor pembrolizumab at 200 mg/3 weeks, with the first dose on 2021-9-19.

Six months after cryoablation, the patient had received a total of 6 cycles of immunotherapy. At that time, the imaging follow-up revealed that the ablated area in the upper lobe of the left lung had become smaller and firmer. Amazingly, the previous solid nodules of varying sizes in both lungs had almost disappeared, especially the larger mass in the lower lobe of the right lung, which was approximately 30 mm in diameter, was found to have largely vanished with only a tiny amount of cords remaining on this examination. In addition, a small amount of fluid in bilateral chest cavities had been wholly absorbed (**Figures 2E–H**).

At the subsequent three-month follow-up, we found that the residual scar in the area of cryoablation in the upper lobe of the left lung had further shrunk, the entire lung was free of any new or enlarged lesions than before, and the residual cords in the original larger tumor area in the lower lobe of the right lung had disappeared entirely (**Figures 2I–L**). At this point, we believe that cryoablation followed by the PD-L1 inhibitor has a powerful synergistic anti-tumor effect that stops tumor progression and even provides a complete cure for the tumor. And the patient strongly proved our hypothesis. To date, the patient has undergone a total of 12 cycles of immunotherapy, and no significant toxic side effects occurred in the patient after immunotherapy. The patient is continuing immunotherapy, and the impact of treatment is regularly evaluated by biochemical indicators and imaging. No signs of tumor recurrence or metastasis have been detected nearly 1 year after cryoablation, and the clinical efficacy is assessed as complete response (CR).

## Discussion

This study is an encouraging report of a case of cryoablation in synergy with PD-1/PD-L1 pathway inhibitor pembrolizumab against OCCC metastasis. The patient underwent surgery for ovarian cancer for 5 years, followed by a period of multiple cycles of chemotherapy. However, the tumor still progressed systemically, and then cryoablation combined with immunotherapy at our institution achieved a surprising outcome that we did not expect, with complete clearance of tumors of different sizes in the lung, confirming the incredible synergistic anti-tumor effect between cryoablation and PD-1/PD-L1 pathway inhibitors.

Unlike thermal ablation, such as radiofrequency ablation and microwave ablation, cryoablation results in the formation of large amounts of *in situ* tumor-associated antigens (TAA), which can help the body achieve *in situ* immunity, mainly through the presentation of Dendritic cells (DC), which induce the production of tumor-specific CD8+ T lymphocytes in the draining lymph nodes (12) and stimulate the host immune system to develop anti-tumor immune effects against primary and metastatic tumors (9, 13), resulting in the regression of the primary tumor and distant metastases, an additional effect known as the “abscopal effect” (14), which was believed to be the basis for synergistic anti-tumor effects with immunotherapy (15–17). Kumar et al. performed palliative cryoablation of breast lesions in a 68-year-old female with lymph node metastases. At 10-month postoperative follow-up, not only was the primary lesion completely eliminated, but the metastatic lymph nodes were also significantly reduced, and their metabolic activity was diminished considerably, demonstrating the “distal effect” caused by cryoablation (16).

Tumor immunotherapy is a method to control or even destroy tumors by using antigens, antibodies, vaccines, cells, or viruses to actively or passively alter the function of the host immune system and cause the body to generate or enhance the anti-tumor immune response (18). Immune checkpoint blockade therapy is currently considered the most promising immunotherapy approach. Immune checkpoints are molecules on the surface of immune cell membranes that conduct inhibitory expression signals and protect the body from intense autoimmune responses under normal conditions. Still, in the tumor microenvironment, it allows tumor cells to successfully escape the immune system (19). The most widely explored immune checkpoint molecules include cytotoxic T lymphocyte-associated antigen-4 (CTLA-4) and programmed death receptor-1 (PD-1) (19–22). By blocking the corresponding signaling pathways, the activation and proliferation of T cells can be restored, and the body’s anti-tumor immune response can be enhanced (23). For instance, Wang et al. reported that the combination of anti-CTLA-4 and anti-PD-1 antibodies significantly inhibited the proliferation of gastric cancer MKN-45 and MGC-803 cells, induced apoptosis, and inhibited tumor cell migration, invasion, and invasion of epithelial-mesenchymal transformation, demonstrating sound anti-tumor effects (24).

This study treated patients with the PD-1/PD-L1 pathway inhibitor pembrolizumab, which was used after cryoablation of a single lesion and demonstrated a strong systemic anti-tumor immune response. This strongly confirms the currently promoted hypothesis that cryoablation and PD-1/PD-L1 pathway immune checkpoint inhibitors have potent synergistic anti-tumor effects. Zhu et al. used PD-1 inhibitors combined with cryoablation to treat renal cell carcinoma in mice. They found that the combined treatment inhibited metastatic growth significantly more than administration of the drug alone or cryoablation alone and that PD-1 inhibitors had a significantly enhanced effect on Cryoablation-induced immune response (including an increased number of CD8+ TIL and increased mRNA expression levels of INF-γ and GZMB) (25). Inhibitors of the PD-1/PD-L1 pathway are currently the first-line treatment for non-small cell lung cancer with >50% PD-L1 expression in tumor tissues and no oncogenic molecular target identified. Still, in most clinical trials, only about a 20% response rate was observed, which may be related to the low number of tumor-infiltrating lymphocytes (TIL) in the tumor microenvironment (26, 27). The TPS value in PD-L1 expression in the patient’s tumor tissue in this study was only 5%, which was far from the criteria for applying PD-L1 inhibitors in the first line. Still, the application after cryoablation demonstrated a strong anti-tumor immune response, which directly led to the regression of multiple tumors in the whole body. Combined with previous studies, we suggest that it may be related to a cryoablation-induced increase in PD-L1 expression in distant tumor tissues, which facilitates antibody binding to PD-L1 in tumor tissues and activates the body’s anti-tumor immune response. In a recent animal experiment we are conducting, we also confirmed that cryoablation induced an increase in PD-L1 expression in distant tumors. The results of this part of the research are currently in submission.

## Conclusion

In conclusion, we inspiringly report a case of cryoablation followed by the application of PD-L1 inhibitor to treat multiple systemic metastases from ovarian clear cell carcinoma. The combination of the two miraculously eradicated solid tumors of different sizes. Although the exact mechanism of action has not been explored, we have reason to believe that the combination of cryoablation and immune checkpoint inhibitor has a powerful synergistic anti-tumor effect, which is yet confirmed by more basic research and clinical applications in the upcoming future.

## Data availability statement

The raw data supporting the conclusions of this article will be made available by the authors, without undue reservation.

## Ethics statement

Written informed consent was obtained from the individual for the publication of any potentially identifiable images or data included in this article.

## Author contributions

LM, ZZ, and YX conceived and designed the study. LM and ZZ drafted the entire manuscript. XiaZ and XBZ are responsible for patient information acquisition and postoperative follow-up. XX and XH are responsible for imaging and image processing. YW and XinZ reviewed and revised the manuscript. BW and ZZ participated in figures and table preparation. YX guided the article revision. All authors contributed to the article and approved the submitted version.

## Funding

This work was supported by the National Natural Science Foundation of China (Grant number: 81771944).

## Conflict of interest

The authors declare that the research was conducted in the absence of any commercial or financial relationships that could be construed as a potential conflict of interest.

## Publisher’s note

All claims expressed in this article are solely those of the authors and do not necessarily represent those of their affiliated organizations, or those of the publisher, the editors and the reviewers. Any product that may be evaluated in this article, or claim that may be made by its manufacturer, is not guaranteed or endorsed by the publisher.
